# Comparative Studies on the pH Dependence of D_OW_ of Microcystin-RR and -LR Using LC-MS

**DOI:** 10.1100/tsw.2011.17

**Published:** 2011-01-05

**Authors:** Gaodao Liang, Ping Xie, Jun Chen, Ting Yu

**Affiliations:** ^1^ Donghu Experimental Station of Lake Ecosystems, State Key Laboratory of Freshwater Ecology and Biotechnology of China, Institute of Hydrobiology, Chinese Academy of Sciences, Wuhan, China; ^2^ Wuhan Centers for Disease Prevention and Control, Wuhan, China

**Keywords:** mcrocystins, n-octanol/water distribution ratio, pH

## Abstract

Microcystins (MCs) are well known worldwide as hepatotoxins produced by cyanobacteria, but little is known about the physicochemical properties of these compounds. The dependence of the n-octanol/water distribution ratio (D_OW_) of MC-RR and -LR to pH was measured by high-performance liquid chromatography combined with mass spectrometry (LC-MS). There was a remarkable difference in such relationships between MC-RR and -LR. The log D_OW_ of MC-LR decreased from 1.63 at pH 1.0 to -1.26 at pH 6.5, and stabilized between -1.04 and -1.56 at a pH of 6.5~12.0; log D_OW_ of MC-RR varied between -1.24 and -0.67 at a pH of 1.00~4.00, and stabilized between -1.20 and -1.54 at a pH of 4.00~12.00. The difference of hydrophobicity in acidic condition between MC-RR and -LR is important, not only for the analytical method of both toxins, but perhaps also for understanding the difference of toxicity to animals between the two toxins.

## INTRODUCTION

Microcystins (MCs) produced by cyanobacteria (also known as blue-green algae) occur worldwide[1,2,3], and have been known as potent liver toxins and tumor promoters[4,5,6]. Furthermore, MCs have been regarded as one of the main factors for the high incidence of human liver tumors in southern China[7,8]. The International Agency for Research on Cancer (IARC) has now classified MC-LR into group 2B, i.e., MC-LR is possibly carcinogenic to humans[9]. The universal structure of MCs is cyclical (-D-Ala-L-X-D-erythro-methylAsp-L-Z-Adda-D-Glu-N-methyldehydro-Ala), where X and Z can be variable L-amino acids and different combinations of amino acids form different MCs. So far, more than 70 structurally different MCs have been identified, among which MC-LR and -RR are the most familiar, especially in Asia. X and Z are leucine and arginine for MC-LR, while X and Z are both arginine for MC-RR[10]. The chemical structures of MC-LR and -RR are shown in Fig. 1.

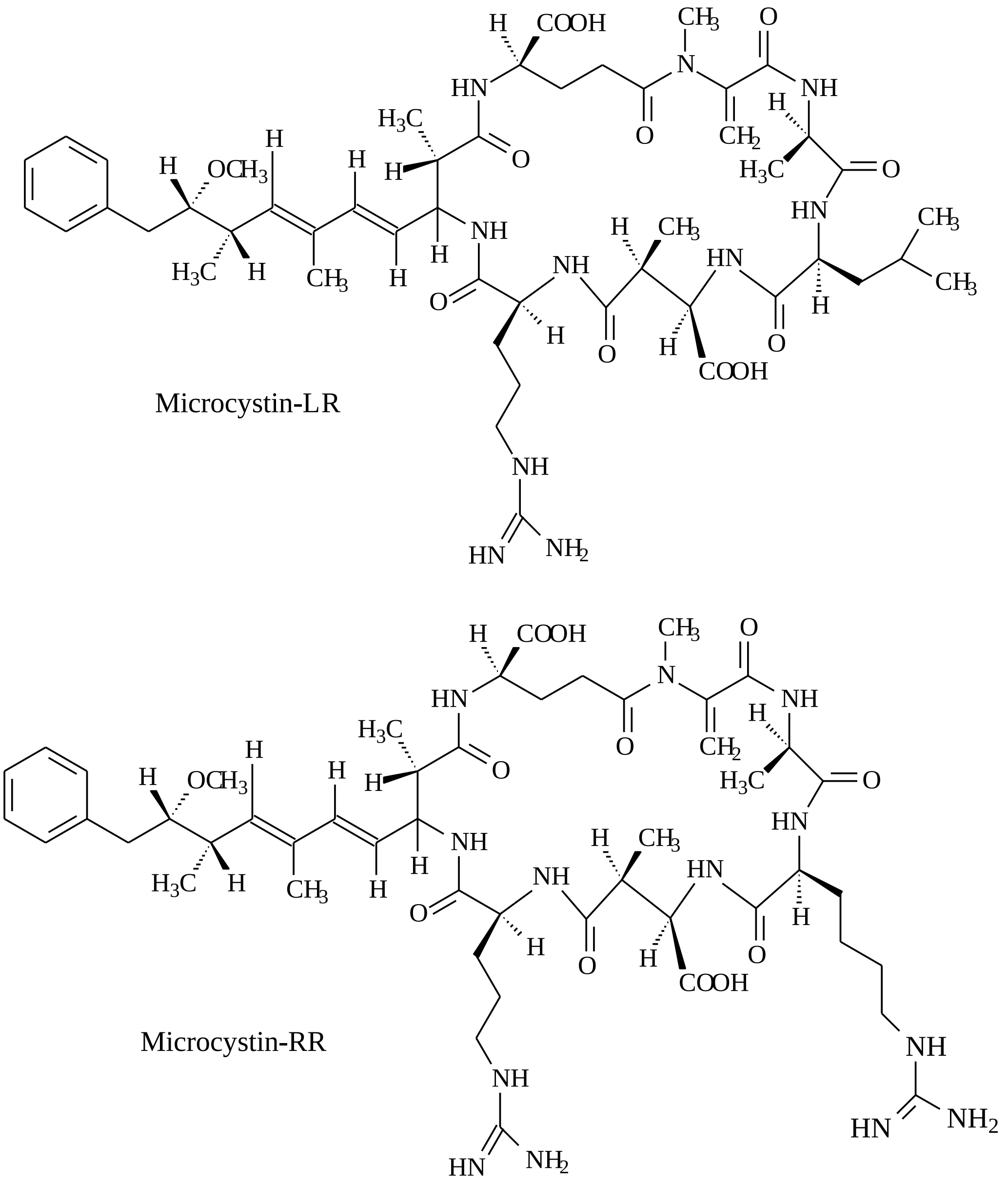

**FIGURE 1.** Chemical structures of MC-LR and -RR.

Although there have been extensive studies on ecotoxicological and human health aspects of MCs, little is known about their physicochemical properties, such as n-octanol/water partition coefficient ($K_{OW}$), vapor pressure of the solid substance ($P_S$) or the subcooled liquid ($P_L$), the aqueous solubility of the solid ($S_S$) or the subcooled liquid ($S_L$), etc.[3,10,11]. Environmental and biochemical behaviors of a contaminant can be predicated by its physicochemical properties[12]. It is well known that $K_{OW}$ is a fundamental property of a contaminant[13,14]. Except the significance for environmental and biochemical behaviors of a contaminant, pH dependence of $K_{OW}$ is very important for the development of an analytical method for ionizable compounds, such as selection of solvent for extraction and decision of pH of mobile phase.

There are two ionizable carboxyl groups and two amino groups in MC-RR, which are not part of the main molecule cycle, while there are two carboxyl groups and one amino group in MC-LR. So it is impossible to procure $K_{OW}$ of these MCs in a neutral molecule directly. Since there are several species of MC in water because of ionizable groups, it is very difficult to measure the distribution of these species in water, and the only available representation of $K_{OW}$ of MCs is the n-octanol/water distribution ratio ($D_{OW}$)[15].

De Maagd et al.[15] measured $D_{OW}$ of MC-LR at different pHs directly by high-performance liquid chromatography (HPLC) and found that log $D_{OW}$ of MC-LR decreased continuously with pH increasing from 1 to 12, which indicates a low tendency of organisms to bioconcentrate MC-LR from water. Due to relatively low sensitivity of HPLC, confidence limits of log $D_{OW}$ became quite large when log $D_{OW}$ was below 1.5 in their experiment. Rivasseau et al.[16] measured the retention factors eluted with water ($K_W$) of MC-LR, -RR, and –YR to estimate their hydrophobicity indirectly, and the log $K_W$ of MCs at pH 7.0 was obtained by graphic extrapolation of some curves, and all were close to 4. Such high hydrophobicity of MCs is quite different from the result of De Maagd et al.[15].

This work aims to measure $D_{OW}$ of MC-RR and -YR in various pHs by liquid chromatography combined with mass spectrometry (LC-MS), and to compare the difference in pH dependence of $D_{OW}$ of these two toxins.

## EXPERIMENT

A solution of NaCl-water (9 g/L) was prepared for various pHs, and the pH of this solution was adjusted to between 1.00 and 12.00 with a step of 0.5 by adding a solution of HCl with appropriate concentration or a solution of NaOH with appropriate concentration. The pH was defined by a pH meter equipped with a pH/ATC multiplex electrode (PB-10, Sartorise), and 1 µL MC-RR in methanol (about 1000 mg/L, extracted from cyanobacteria powder and purified with semi-prep-LC [Waters 600, USA] and solid phase extraction [SPE] cartridges by ourselves, and the ultimate product was identified by comparing the MS/MS spectrum with that of commercial standard samples and checked by HPLC with a purity of over 95%) and 3 µL MC-LR (about 400 mg/L, obtained by the same process with that of MC-RR) were added into a 10-mL glass centrifugation tube in which a 1.00-mL NaCl solution at different pH was added previously. One milliliter of n-octanol (A.R.) was added to each tube. Then the tubes were shaken (300 rpm) by a shaking bed (ZHWY-100B, Shanghai Zhichenng Instrument Company) for 12 h, while the temperature of the bed was stabilized at 37 ± 1℃. Subsequently, the tubes were centrifuged for 10 min at 1500 rpm (LXJ-II, Shanghai Medical Analytical Instrument Company).

Of the n-octanol phase, 0.7 mL was transferred into another centrifugation tube. The rest of the n-octanol and some water on the top (about 0.3 mL) were discarded. The remaining water was transferred into a 1.8-mL sample vial and analyzed directly. Then, 0.7 mL water (pH = 12) was added into the 0.7-mL n-octanol phase. The tube was shaken and centrifuged with the same condition described above. The n-octanol phase and some water from the top were discarded. The remaining water was transferred into a 1.8-ml sample vial and analyzed. Triplicate treatments were performed at pH = 5.00, 6.00, 6.50, 9.00, and 9.50, while only a single treatment was done at other pHs.

To measure the concentration of MCs in water and the n-octanol phase, a method based on HPLC-ESI-MS/MS technology was developed. A Surveyor HPLC system (Thermo Electron Corporation, USA), including a Surveyor LC pump, a Surveyor autosampler, and a Surveyor photoelectric diode array (PDA) detector, was combined with a LCQ Advantage MAX mass spectrograph (Thermo Electron Corporation) equipped with an Electrospray ionization probe to analyze the sample prepared previously. A Hypersil Gold column (2.1-mm i.d.*150-mm length, 5 µm, Thermo Electron Corporation) was applied. Solution A of the elution system was a formic acid (p.a.)-water solution (0.05%, v/v), while solution B was a formic acid-acetonitrile (TEDIA Company, HPLC grad) solution (0.05%, v/v). The elution program was described as follow: percentage of solution B was initialized 25% for 2 min, subsequently increased to 50% in 5 min linearly, and then increased to 100% in 4 min with no stay. Then it was held at 100% for 4 min to end an analysis cycle. The total flow rate was held at 0.2 mL/min at the analysis stage. The percentage of solution B was adjusted to 25% and the flow rate was increased to 0.3 mL/min for 5 min before the next injection to renew the initial condition rapidly. The injection volume was 10 µL. The temperatures of the vial tray and column oven in the autosampler were set to 10 and 25℃, respectively. The divert valve integrated on the mass spectrograph was set to waste in the initial 3 min to avoid the spectrograph being contaminated by salt in sample solution. MS was set to ESI+ mode, and MS tuning and optimization were achieved by infusing MC-RR with ion of $[M+2H]^{2+}$ at m/z of 520. Quantification of MCs was achieved through total signal of MS/MS. Precursor ion was $[M+2H]^{2+}$ at m/z of 520 for MC-RR, while precursor ion was $[M+H]^{+}$ at m/z of 995.5 for MC-LR. Collision energy was 37% for both MC-RR and -LR.

The area of the chromatographic peak representing MC-RR or -LR in the water phase sample was divided by the corresponding peak area of MC-RR or -LR in the n-octanol phase directly; thus $D_{OW}$ with its logarithm at different pH was calculated.

The flow chart of this experiment is shown in Fig. 2.

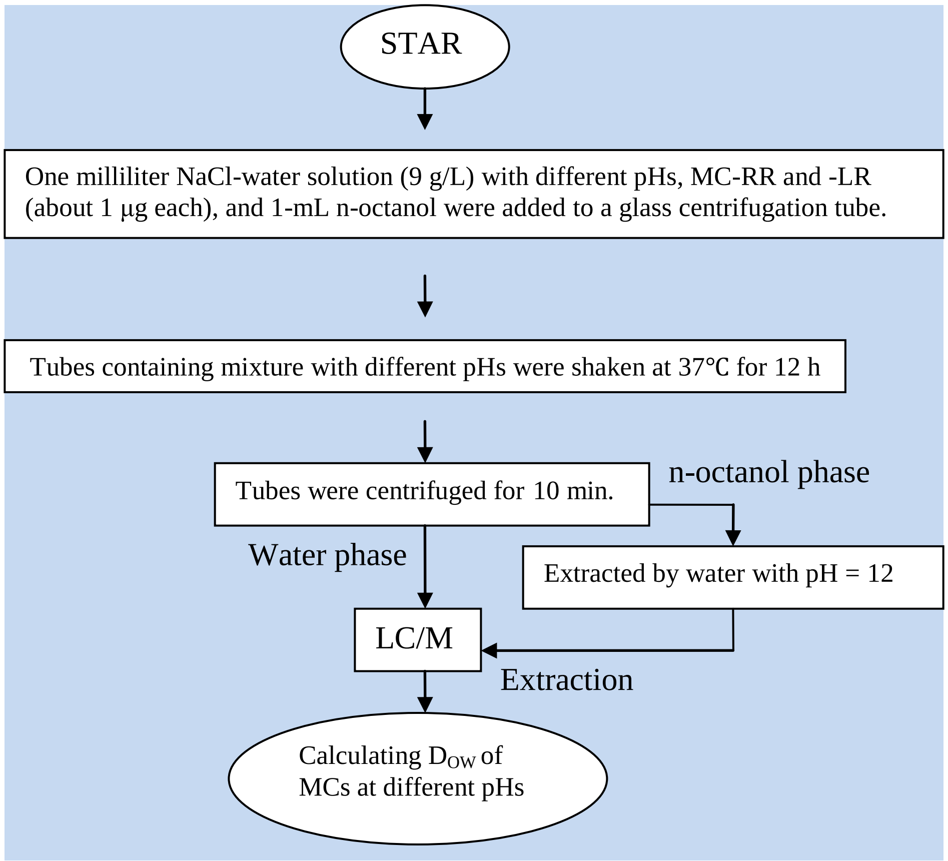

**FIGURE 2.** Experimental flow chart.

## RESULT AND DISCUSSION

To evaluate the method, linearity and limit of detection were estimated for both MC-RR and -LR, and the recoveries of back extraction from n-octanol into water (pH = 12) were also defined. Limits of detection (LOD) were 2 and 0.7 µg/L for MC-RR and -LR, respectively. The correlation coefficient ($r^2$) values for the calibration curves (with six concentrations from 10 to 2500 µg/L each) were both >0.99. All concentrations of MCs in this study were between 10 and 1000 µ/L (all $D_{OW}$ were between 0.01and 100, and the initial concentration was 1000 µ/L), so these concentrations could be measured accurately by this method. The recoveries were 97.9 ± 8.4% and 100 ± 7.3% (n = 3) for MC-RR and –LR, respectively, with spiked concentrations of both 10 µg/L, and the values were 99.5 ± 5.8% and 97.6 ± 5.5% with spiked concentrations of both 1000 µg/L. Because all the recoveries of back extraction covered 100% with statistical significance, no correction was needed.

pH~log $D_{OW}$ curves of MC-RR and -LR are shown in Fig. 3. Different from the result of De Maagd et al.[15], log $D_{OW}$ of MC-LR in the present study was almost constant between pH 4 and pH 12, and decreased sharply from 1.59 at pH 2 to -1.20 at pH 6, similar to other ionic compounds, such as pentachlorophenol[17]. Values of log $D_{OW}$ of both MC-LR and -RR were constant between pH 4 and pH 12, probably because of the ion-pair effect that can stabilize $D_{OW}$ via ion pairs. The concentration of NaCl used by De Maagd et al. was lower than that used in the present study, so the ion-pair effect was not obvious and there was no constant log $D_{OW}$ level observed in the result. Different from MC-LR, the log $D_{ow}$ of MC-RR varied between -1.24 and -0.67 at a pH of 1.00~4.00, and stabilized between -1.20 and -1.54 at a pH of 4.00~12.00.

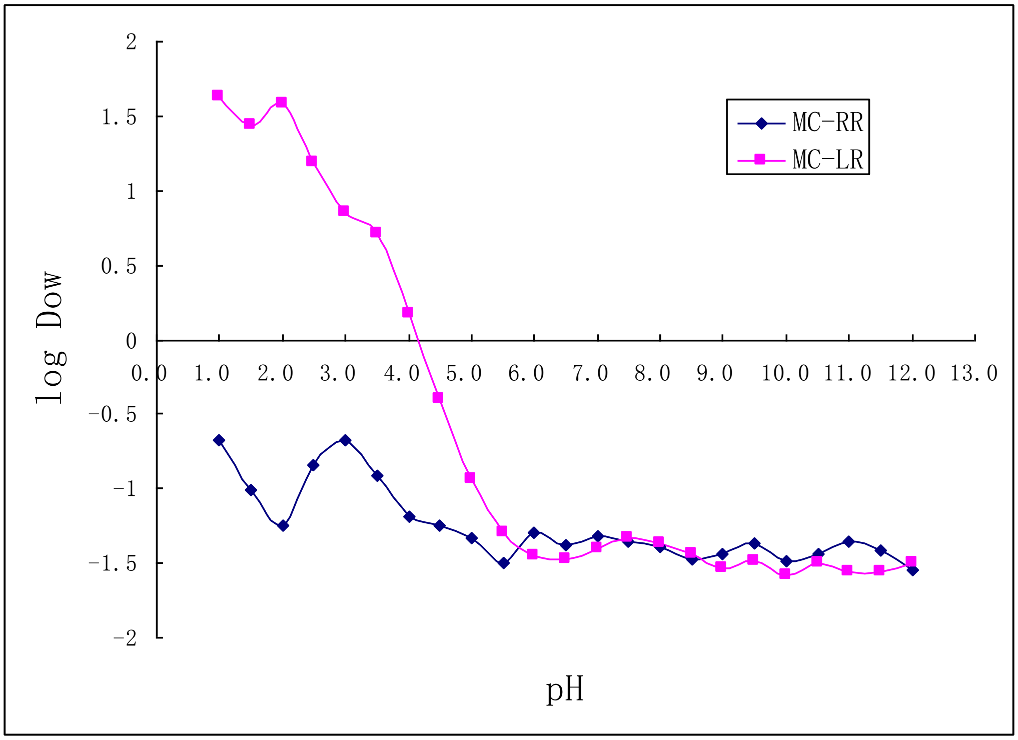

**FIGURE 3.** pH-log $D_{OW}$ curve of MC-RR and –LR.

The difference between MC-RR and -LR indicated that the tendency of MCs to partition water likely relied on the ability of disassociation and ion species of MCs. This was similar to other ionizable compounds[19]. On the whole, the ($COO^-$) ion was more hydrophilic than the ($NH_2^+$) ion. The pKa values of the carboxyl group and amino group were about 2.1 and 12.5, respectively[15]. As shown in Fig. 1, both MC-LR and -RR have two carboxyl groups, so they have equal ability to disassociate into anion and also have similar behaviors in basic condition, and log $D_{ow}$ had lower values when the two carboxyl groups of both MC-RR and -LR were disassociated absolutely (pH > 5.5). This study approved the hypothesis of De Maagd et al.[15]. According to the study of De Maagd et al., the $(COOH)_2(NH_2^+)$ species was more hydrophobic than the $(COO^-)(NH_2^+)$ species. The dominant species was $(COOH)_2(NH_2^+)$ when pH < 2 for MC-RR, which was $(COOH)_2(NH_2^-)_2$ for MC-LR. In addition, the $(COOH)_2(NH_2^+)$ species was not the dominant species at any pH for MC-RR because the two amino groups were disassociated absolutely at these pHs. So MC-RR is much more hydrophilic than MC-LR when pH < 2. Based on the differentia and homology between MC-RR and -LR, the order of hydrophobicity of species could be estimated as $(COOH)_2(NH_2^+)$ >> $(COOH)_2(NH_2^+)_2$ > $(COO^-)(NH_2^+)$ > $(COO^-)(NH_2^+)$ > $(COO^-)_2(NH_2^+)_2$ = $(COO^-)_2(NH_2^+)$. The estimated dominant species of MC-RR and -LR at different pHs are show in Table1. This may be the main reason for the difference in hydrophilia between MC-RR and -LR in the present study. There were similar valleys in both curves of MC-LR and -RR in the acidic zone, probably because there were two stages in disassociation of the two carboxyl groups.

**TABLE 1**
**Dominant Species of MC-RR and -LR at Different pHs**

| pH | pH < 2 | 2 < pH < 5.5 | pH > 5.5 |
|---|---|---|---|
| MC-RR | $(COOH)_2(NH_2^+)_2$ | $(COO^-)(NH_2^+)_2$ | $(COO^-)_2(NH_2^+)_2$ |
| MC-LR | $(COOH)_2(NH_2^+)$ | $(COO^-)(NH_2^+)$ | $(COO^-)_2(NH_2^+)$ |

Cyanobacterial blooms occur normally at a pH between 6 and 9[18], where both MC-RR and -LR are comparatively hydrophilic. This suggests a low tendency of aquatic animals to bioaccumulate dissolved MCs from water, but many researches support bioconcentration of MCs in laboratory and field conditions[20,21,22,23]. Perhaps the difference in pH of the water environment and gastric juices of animals should be taken into account when bioaccumulation of MCs was predicated.

Although the result of this research could partly explain different behaviors between the two most important MCs, MC-LR and -RR, comparison between more MC species are awaited because more details need to be explored, such as the relationship between pH dependence of $D_{OW}$ and chemical structures. If every $pK_a$ value of the ionizable group could be measured accurately, the percentage of any potential species at any pH could be calculated. When enough $pK_a$ values and pH dependence of $D_{OW}$ are measured, the effect of chemical structures to pH dependence of $D_{OW}$ could be explored further.

## ACKNOWLEDGMENTS

This study was jointly supported by the National Natural Science Foundation of China (30623001)and National Basic Research Program of China (973 Program) (2008CB418101).
